# Elevated eosinophil levels observed in infantile hemangioma patients from Kaifeng, China

**DOI:** 10.12688/f1000research.21608.1

**Published:** 2019-12-16

**Authors:** Xianglei Li, Chunyan Ma, Jiaoyang Xu, Biao Gao, Michael Steele, Adi Idris

**Affiliations:** 1 Department of clinical laboratory, Kaifeng Central Hospital, Kaifeng, Henan province, China; 2 Faculty of Health Sciences, Australian Catholic University, Brisbane, Queensland, Australia; 3 Menzies Health Institute Queensland and School of Medical Science, Griffith University, Gold Coast, Queensland, Australia

**Keywords:** Infantile hemangioma, eosinophil, haematology, China

## Abstract

Infantile hemangioma (IH) is one of the most common soft-tissue neoplasms of infancy. Although clinical diagnosis for IH is well-established, the haematological parameters associated with IH are not well explored. In this short study, we observed significantly higher eosinophil (EO) numbers in IH patient blood compared to healthy controls. This contributed to the observed higher EO % in the peripheral blood of IH patients and was irrespective of age. This new haematological finding could carry a potential diagnostic/prognostic relevance for IH.

## Introduction

Infantile hemangioma (IH) is a common benign tumour in children that presents as precursor vascular lesions, which either present at birth or develop during the early neonatal period and undergo rapid proliferation ^1^. IH is the most common vascular tumour of infancy, occurring in up to 10% of infants ^2^ and is characterized by high expression of genes involved in vasculogenesis, angiogenesis and tumorigenesis ^3^. In the Chinese population, low birth weight, prematurity and maternal progesterone have been associated with IH development ^4^. Although clinical diagnosis for IH is well-established, other than the proposed embryonic stem cell origins of IH ^5^, little is known about the peripheral blood cell repertoire in IH patients, let alone in Chinese patients. This concise study seeks to determine any potential haematological signature(s) that may be present in the peripheral blood of IH diagnosed Chinese patients. In this retrospective study, we report significantly elevated eosinophil numbers in Chinese IH patients.

## Methods

Kaifeng Central Hospital (Kaifeng, China) is designated as a health care centre by the Kaifeng city government. Retrospective analysis of Kaifeng Central Hospital patient records was performed for this study and the study protocol was approved by the Kaifeng Central Hospital Ethics Committee, which waived the need for informed consent from patients/guardians for the use of their records. Underlying data are all de-identified demographic variables and blood parameters for each individual patient ^6^. Patients’ parents/guardians had been made aware that this data could be used for research purposes.

Study subjects included paediatric patients (n = 1631) of all sexes (Male (M) = 460 / Female (F) = 1171) between the ages of 0 to 12 months (3.77 ± 2.98 months, mean ± SD) who were diagnosed with IH from January 2011 to December 2016. Control subjects (n=1602) were healthy children who had blood taken during routine medical check-up visits to the hospital during that same period. As previously seen ^7^, we observed significantly more female IH patients than males (Chi squared test, p<0.001). The inclusion criteria included only infants up to 12 months of age and infants with all variables measured (WBC, RBC, MPV, HGB, PCT, EO%, EO#). The exclusion criteria were subjects with other existing conditions and diseases including eczema, systemic infection, allergy, haematological diseases, immunological diseases and adrenocortical insufficiency and who were not undergoing treatment for IH.

Peripheral blood samples (n = 3233) were assayed for full blood panel count on the Sysmex XN-800i (Sysmex Europe GmbH, Norderstedt, Germany) as per manufacturer’s protocol. Blood variables measured included white blood cell (WBC) counts, red blood cell (RBC) counts, mean platelet volume (MPV), haemoglobin (HGB) levels, procalcitonin (PCT) levels and eosinophils (EO) percentage/counts.

Due to strong non-normality of some variables the non-parametric Mann-Whitney Test was used in the analysis of continuous variables. Chi-Square test of independence was used for categorical data. All statistical analysis was done on IBM SPSS Statistics 22.0 (SPSS Institute, Chicago, IL, USA). Before analysis, all variables were reviewed for accuracy of data entry and missing values. Due to the large sample size involved, statistical analysis is focused primarily on frequencies and percentages.

## Results

We analysed blood parameters between IH patients and healthy controls ( Table 1). Notably, we observed a high elevation of EO numbers in IH patients compared to healthy subjects. Compared to the healthy control (0.19±0.24 ×10 ^9^/ µL), there is an almost significantly (Chi-Square test of independence, p<0.001) two-fold higher EO count in IH patients (0.4±0.37 ×10 ^9^/ µL). This contributed to the observed higher EO % in the peripheral blood of IH patients.

**Table 1.** Haematological profile in healthy and infantile hemangioma subjects.

	IH (n=1631)	Control (n=1602)	P-value
Age (in months) Mean (SD)	3.77 (2.98)	3.44 (2.67)	0.016 ^1^
Median (IQR)	3 (5)	2 (4)	
0–3 months N (%)	958 (50.0)	958 (50.0)	
4–6 months N (%)	364 (50.4)	358 (49.6)	
6–12 months N (%)	309 (51.9)	286 (48.1)	
Gender Male N (%)	460 (38.5)	736 (61.5)	<0.001 ^2^
Female N (%)	1171 (57.5)	866 (42.5)	
WBC (10 ^9^/µL)	10.10 (3.21)	10.39 (4.38)	
RBC (10 ^6^/µL)	4.33 (0.69)	4.79 (0.51)	
MPV (fL)	9.74 (0.81)	9.47 (0.81)	
HGB (g/L)	113.59 (16.90)	122.74 (14.78)	
PCT (%)	0.05 (0.03)	0.04 (0.02)	
EO %	3.96 (2.46)	1.91 (2.11)	<0.001 ^1^
EO # (10 ^9^/ µL)	0.40 (0.37)	0.19 (0.24)	<0.001 ^1^

Control – Healthy subjects, IH-Infantile hemangioma patients, WBC- white blood cells, RBC- Red blood cells, MPV – Mean platelet volume, HGB- Hemoglobin, PCT-Procalcitonin, EO-Eosinophils ^1^ Mann-Whitney Test ^2^ Chi-Square Test of Independence

This observation was irrespective of age as significantly higher EO numbers (Mann-Whitney test, p<0.001) were observed only between IH patient and healthy control cohort for each age-matched group, not between each age group ( Table 2). Other measured blood parameters were comparable between IH patients and healthy controls ( Table 1).

**Table 2.** Comparison of the levels and percentage population of eosinophils among different age groups between healthy and infantile hemangioma subjects.

	IH mean (SD)	Control mean (SD)	P-value
Aged 0–3 months EO %	3.90 (2.47)	1.88 (2.18)	<0.001 ^1^
EO #	0.42 (0.36)	0.19 (0.25)	<0.001 ^1^
Aged 4–6 months EO %	4.28 (2.64)	1.84 (1.84)	<0.001 ^1^
EO #	0.42 (0.45)	0.19 (0.22)	<0.001 ^1^
Aged 7–12 months EO %	3.77 (2.15)	2.10 (2.17)	<0.001 ^1^
EO #	0.34 (0.23)	0.20 (0.22)	<0.001 ^1^

^1^ Mann-Whitney Test

## Discussion

Elevated EOs are classically associated with the presence of inflammation in patients with conditions such as asthma, allergy and parasitic infections. Our exclusion criteria in this study discounted any possibility of this on our observations. Previous haematological analyses of blood collected from 34 IH patients in an Italian study revealed slightly elevated EO % ^8^, but IH blood parameters were not compared to that in healthy subjects. Mean EO reference numbers in the general Chinese population are between 0.1 – 0.2 × 10 ^9^ ^9^, in concordance with healthy EO levels we observe.

One major limitation in this study is the inability to compartmentalize IH patients into different clinical phases (i.e. proliferating phase, early regressing (involuting) phase, and advanced regressing (involuted) phase) as this information was not made available to us during retrospective data collection. Future work will focus on determining whether EO numbers increase progressively throughout the different IH clinical phases.

Propranolol, a beta-blocking agent, has been used as the first-line therapy for the management of IH since 2008 ^10^. However, propranolol use for managing IH in China only came about after findings from a prospective 2011 trial ^11^. Given that propranolol has been shown to prevent the release of EO-activating cytokines ^12^, propranolol would work favourably in IH patients to reduce the abnormally high EO numbers seen in our patients. In this present study, we show for the first time a significant elevation in EO numbers in IH paediatric patients and this could potentially carry a diagnostic/prognostic relevance in Chinese children. IH is commonly diagnosed clinically based on natural history of the lesion. Currently, the most important marker to accurately diagnose IH is glucose transporter 1 (GLUT1) ^13^, though this marker is present despite the proliferative activity of the IH lesion ^14^. The use immune cytokines as a potential biomarker for IH progression was recently proposed ^8, 15^ and some of those cytokines (e.g. interleukin -8) could directly impact EO proliferation. Standard haematological (e.g. abnormal EO numbers) and unique cytokine signatures could potentially serve as a diagnostic/prognostic marker for IH progression.

## Data availability

Open Science Framework: Elevated eosinophil levels observed in infantile hemangioma patients from Kaifeng, China, https://doi.org/10.17605/OSF.IO/P8XR3 ^6^.

This project contains the following underlying data:

- raw data_Li *et al.*xls (Raw haematological data)

Data are available under the terms of the Creative Commons Zero “No rights reserved” data waiver (CC0 1.0 Public domain dedication).
