# Supplementary material for: Elevated eosinophil levels observed in infantile hemangioma patients from Kaifeng, China
Source: F1000Res. 2019 Dec 16;8:2111. [Version 1] doi: 10.12688/f1000research.21608.1 (PMC6993820; doi:10.12688/f1000research.21608.1)
Supplement: Peer review history

# Peer review: Reviewer response for version 1

**DOI:** 10.5256/f1000research.23816.r57885

**Published:** 2020-01-27

**Authors:** Swaminathan Sethu

**Affiliations:** 1 GROW Research Laboratory, Narayana Nethralaya Foundation, Bangalore, Karnataka, India

The following are minor suggestions that may improve the clarity and interpretation of the data

1. The title can be “Elevated peripheral blood eosinophil levels in infantile hemangioma patients” or “Elevated peripheral blood eosinophil levels in Chinese patients with infantile hemangioma”.
2. The authors have pointed out that IH is significantly higher in females compared to males. It would be useful to analyse and represent the results in table 1 and 2 based on gender. In other words, in addition to the current statistical analysis, it would be interesting to know whether the eosinophil (EO) levels were significantly different between controls and IH in males and females subjects separately. The authors can also expand the Table 1 parameters based on gender as well.
3. Further, it will be useful to know the normal range for EO in pediatric population. The authors have mentioned the range for Chinese adult population. The authors can calculate the proportion (%) of subjects with IH above the normal range (if available) or the proportion (%) of subjects with IH above the median level in the control group. The authors can also attempt AUC analysis, if possible to improve the clinical relevance of EO levels in the diagnosis of IH.
4. It would be useful to know whether the authors had access to the proportions of other leukocyte subsets other than EO. This would be relevant and the authors can consider addressing this in the discussion.
5. It is unclear how the % of Procalcitonin (PCT) was computed and whether it is possible to include the concentration range for the same. Further, it was not stated as to why PCT was included in the inclusion criteria and how it is relevant to IH or EO levels in the context of IH.
6. Could power calculation be done to show the robustness of the finding with relevance to the sample size?

I confirm that I have read this submission and believe that I have an appropriate level of expertise to confirm that it is of an acceptable scientific standard.

# Peer review: Reviewer response for version 1

**DOI:** 10.5256/f1000research.23816.r57886

**Published:** 2020-01-02

**Authors:** Mark I.R. Petalcorin

**Affiliations:** 1 PAPRSB Institute of Health Sciences, Universiti Brunei Darussalam, Gadong, Brunei

In this concise article, Li and co-authors reported that the infantile hemangioma (IH) patients showed significantly higher eosinophil levels more than the healthy control subjects suggesting a potential diagnostic relevance. Although promising, the article requires more in-depth analysis especially in addressing the causation factors that result in the increase of eosinophil levels.

 I have some concerns on the data analysis. Firstly, the mean values of percent eosinophils observed in IH patients of 3.96 and in healthy subjects of 1.91, as shown in Table 1, are both still within the normal clinical range of 0-6% EO (Medscape). Thus, the elevated eosinophil levels in IH patients might be interpreted as physiologically irrelevant by clinicians as the values are still within the normal range.

 Secondly, whilst the difference of %EO values between IH and healthy subjects is statistically significant, the raw data show that only 15% of the total IH patients and about 5% of the healthy subjects have high %EO values above the normal range of 6%. It would be useful if the authors will include this in their analysis considering the clinical implications. In addition, the authors should also specify what is the normal range of %EO used in China as this can vary in different clinical laboratories.

 Lastly, I think that there are other factors contributing to the observed increase of eosinophil levels that might be present but not measured in this study such as drug treatment given to IH patients, which could be the underlying cause of the increase but not taken into account. This is the limitation of a retrospective study such as this, in which the authors have no control on how the data were designed and collected, and whether the patients received drug treatment or not. The claim of a potential diagnostic usefulness for this study is an overestimation as the causation is not well-established but only through the association. But this is a good pilot study to test the hypothesis that can be further explored.

 As a minor comment, it would be useful to include the units used for all the  parameters mentioned in the raw haematological data.

I confirm that I have read this submission and believe that I have an appropriate level of expertise to confirm that it is of an acceptable scientific standard.
